# On residual stresses and homeostasis: an elastic theory of functional adaptation in living matter

**DOI:** 10.1038/srep24390

**Published:** 2016-04-26

**Authors:** P. Ciarletta, M. Destrade, A. L. Gower

**Affiliations:** 1 Sorbonne Universités, UPMC Univ Paris 06, CNRS, UMR 7190, Institut Jean Le Rond d’Alembert, F-75005 Paris, France; 2 MOX and Fondazione CEN, Politecnico di Milano, P.za Leonardo da Vinci 32, 20133 Milano, Italy; 3 School of Mathematics, Statistics and Applied Mathematics, National University of Ireland Galway, University Road, Galway, Ireland; 4 School of Mathematics, University of Manchester, Oxford road, Manchester, M13 9PL, UK

## Abstract

Living matter can functionally adapt to external physical factors by developing internal tensions, easily revealed by cutting experiments. Nonetheless, residual stresses intrinsically have a complex spatial distribution, and destructive techniques cannot be used to identify a natural stress-free configuration. This work proposes a novel elastic theory of pre-stressed materials. Imposing physical compatibility and symmetry arguments, we define a new class of free energies explicitly depending on the internal stresses. This theory is finally applied to the study of arterial remodelling, proving its potential for the non-destructive determination of the residual tensions within biological materials.

Modern physiology developed from the idea that life phenomena result from the mutual equilibrium between the living matter and the surrounding media. First proposed by Claude Bernard^1^, such a dynamic balance was hypothesized to regulate living organisms despite the structural complexity of the constituting matter^2^. Although based on novel experimental observations, this seminal concept somewhat reformulated the old philosophical concept of *harmony* by Heraclitus and Empedocles, as the principle guiding constant change in all beings. Accordingly, this modern conception was later referred to as *homeostasis*, etymologically from the ancient Greek words “homeoios” and “stasis”, roughly indicating the tendency towards a steady state^3^. A network of servo-mechanisms physiologically restores the stable equilibrium between the interior matter of a living entity in the face of external perturbative agents, which are generically identified as *biological stresses*^4^. Although this notion includes stress in the mechanical sense, i.e. the set of physical cues determining a deformation within a body, it is worth noticing that stresses in medicinal sense refer to a much wider ensemble of factors, e.g. anatomical-biochemical and symphato-adrenal stimuli^5^.

Since the pioneering study of Galileo on the allometric scaling of bones in animals with different dimensions^6^, much work has been done to understand the functional adaptation of biological matter to physical forces. Classical examples include the optimal structural remodelling of bone to mechanical loading (Wolff’s law)^7^, and arterial endothelia to shear stress (Murray’s law)^8^. It is now well acknowledged that tensional homeostasis in living matter is regulated through mechano-transduction, i.e. the ensemble of biological processes transforming mechanical cues into biochemical signalling. Living cells sense physical stimuli through integrins and surface receptors, possibly triggering intracellular signalling, which in turn activates the actomyosin contractility to produce internal stresses. Thus, cells balance exogenous and endogenous forces using an iterative process, also known as mechano-reciprocity^9^: in physical terms, this functional adaptation aims to optimize a certain functional, possibly a free energy. Hence, not only can living matter mechanically adapt to the external physical cues, but it can also respond by epigenetic remodelling. This genetic response may drive the onset of pathologies, e.g. solid tumours, expressing malignant genes in response to the higher cytoskeletal tension resulting from an altered stromal interaction^10^. In summary, living materials have the striking ability to change actively their micro-structure for adjusting to the surrounding media, developing a state of internal tension, which even persists after the removal of any external loading. Such *residual stresses*, closely resembling the ones originated by misfits within inert matter^11^, are almost ubiquitous in Nature, being revealed by simple cutting experiments, as depicted in Fig. 1. If a cut produces a measurable opening, which is a revelatory and quantifiable sign of internal tension, it inevitably destroys the material integrity, thus making it unsuitable for determining the complex distribution of residual stresses. Even if non-destructive experimental techniques, e.g. X-ray diffraction and photo-elasticity, have been lately developed in metallurgic research, most of them are either inapplicable to soft living matter or they are only limited to simple geometries^12^. Nonetheless, accounting for the three-dimensional residual stresses is of utmost importance for understanding the properties of many biological materials, since such internal tensions are often of the same order of magnitude as the external loadings, as measured for arteries^13^ or solid tumours^14^.

In this work, we propose a novel constitutive theory for internally stressed living matter, demonstrating how a principle of functional optimality in response to external loads allows determining the complex distribution of residual stresses. In the existing literature, residual stresses in biological materials are modelled using a *virtual stress-free state*, i.e. a collection of the natural configurations around each material point, as if all the tensions exerted by the surrounding matter could be released^15^. A multiplicative decomposition of the deformation gradient is further applied to restore the physical compatibility of the material, following an earlier approach used in elasto-plasticity^16^. Notwithstanding, such a virtual state does not exist in physical reality and must be postulated *a priori*, often providing an oversimplified, unrealistic representation of the residual stresses.

Our approach goes beyond the state of the art since it does not require any virtual state: instead, it considers that the reference configuration of the body is residually stressed, assuming that its free energy explicitly depends on the pre-existing residual stresses.

## The Nonlinear Elastic Model

**Figure 2:**
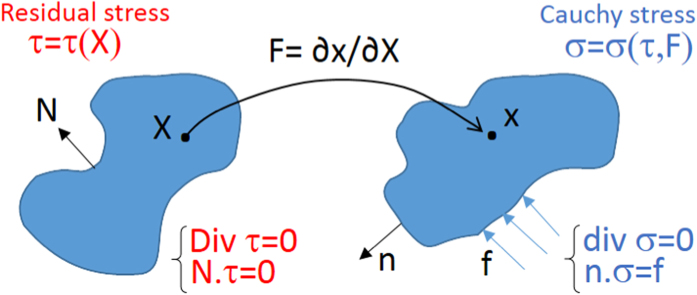
Kinematics of the finite deformation of a residually stressed elastic body subjected to a generic external traction load f in the spatial configuration.

We consider the living body as a continuous distribution of matter at the tissue scale, thus assuming a characteristic length larger than the cell size. Let **X** and **x** be the material and spatial position vectors of a biological material in the reference (residually stressed) and final configurations 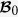 and 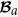, respectively, so that 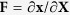 is the geometric deformation gradient, as depicted in Fig. 2.

Furthermore, we choose a characteristic time much larger than the typical time regulating the formation of physical distortions between cells (e.g. few days during embryogenesis^17^), so that the material is initially subjected to a quasi-static distribution of macroscopic residual stresses. Therefore, the body occupies a volume Ω_0_ with a free boundary ∂Ω_0_ with an outward unit normal **N** in the reference configuration, being characterized by a *residual stress tensor **τ***, such that:


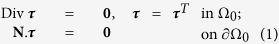


where Div is the material divergence. Therefore, applying the mean value theorem to the equilibrium condition in Eq. (1), we deduce that the residual stress field cannot be uniform within the body, unless it vanishes everywhere, and it must have zero mean value in the reference domain^18^. In addition, we assume that the characteristic time is short enough, with respect to the characteristic dissipation time of the underlying biological bonds, so that the material behaves elastically and a strain energy functional Ψ can be postulated^19^. Disregarding material anisotropy in the following for the sake of simplicity, we further assume that the elastic free energy of the material can be written as Ψ = Ψ(**F**, ***τ***). Applying the representation theorem for tensor functions, Ψ can be written as a scalar function of a set of ten independent invariants^20^: the six principal invariants *I*_*αk*_, with *α* = (*C*, *τ*) and *k* = (1, 2, 3), of **C** = **F**^*T*^**F** and ***τ***, and the following four combined invariants^21^:


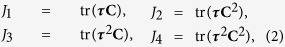


where **tr** indicates the trace operator. Accordingly, the symmetric Cauchy stress tensor ***σ*** = ***σ***(**F**, ***τ***) in the deformed configuration reads (see Supplementary Material for further details):


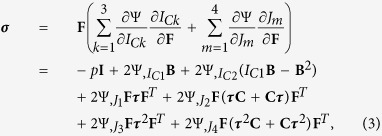


where **B** = **FF**^*T*^, comma denotes partial derivative and *p* is the Lagrange multiplier ensuring the incompressibility constraint det **F** = 1, which generally applies to soft living matter at tissue scale, since they are mostly composed of water^22^. The equilibrium condition imposes that div **σ** = **0** in the spatial domain, div being the spatial divergence, while the value of *p* can be fixed by applying the generic boundary conditions in the spatial configuration, possibly representing external loading and/or imposed displacements.

In order to propose a physically meaningful constitutive relation for Ψ as a function **F** and ***τ***, a number of physical conditions must be imposed. First of all, the free energy must be zero for an unstressed and undeformed body, so that Ψ(**I**, **0**) = 0. Second, we must enforce the *compatibility* of the residual stress, i.e. ***σ***(**I**, ***τ***) = ***τ***, giving rise to three scalar equations. Third, we introduce the *initial stress symmetry*, i.e. the necessity to recover the residually stressed configuration from the loaded state by reversing the mapping:


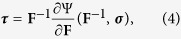


which imposes nine scalar equations involving the derivatives of the free energy with respect to the invariants. Although all such three physical conditions result in a non trivial set of equations, as reported in the Supplementary Material, a great simplification arises if we neglect the functional dependence on *J*_2_, *J*_3_ and *J*_4_. Under these assumptions, we prove that a suitable class of free energies for a residually stressed material has the following functional form:


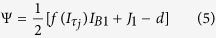


where *d* is a constant and 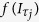 is a scalar function of the residual stress components, which defines their influence on the material response after a superimposed elastic deformation. In particular, we demonstrate that the generalized expression of a neo-Hookean material with residual stresses is given by setting 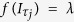 where *λ* is the real positive root of:


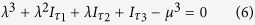


and *d* = 3*μ*, with *μ* being the shear modulus in the absence of residual stresses.

The constitutive relation defined in Eqs (5, 6) defines a novel free energy for finite hyperelastic solids with residual stresses, which is mathematically well-posed thanks to the polyconvexity of Eq. (5). It describes a residually stressed body which physically behaves as a neo-Hookean material, without requiring any guess about the virtual stress-free state, in contrast to the existing approaches based on the multiplicative decomposition. This novel constitutive theory opens a number of applications in the study of the physical behaviour of living matter, especially for the non-destructive experimental determination of the residual stresses. For example, mechanical testing on intact tissues could be used to measure the displacements in controlled loading conditions, which in turn may feed an inverse analysis for determining the initial distribution of the residual stresses. The significance of the proposed theory beyond the existing methods is exemplified in the following applications, concerning the study of the mechanical adaptation of living matter and the non-destructive determination of residual stresses by elastic wave propagation.

## The Functional Adaptation of Arteries

It is well known that when a segment of an artery is cut radially, the sample ring opens to a circular sector, highlighting the presence of residual stresses within the tissue^13^. Since the vessel is internally subjected to the physiological blood pressure, such residual stresses arise to reduce the transmural gradient of the Cauchy stress within the thick tube. Taking the opening sector as the stress-free natural configuration of the artery, it has been hypothesized that residual stresses result from a self-regulatory process reacting to long-term average values of blood pressure towards a homeostatic condition^23^. Although apparently reporting a realistic distribution of opening angles for different physiological conditions and tissue geometries, it has been observed that a radial cutting does not release all the internal tension, and some residual stresses remain. In contrast, we apply the proposed constitutive theory for postulating a principle of functional adaptation regulating the arterial homeostasis through optimal material remodelling.

**Figure 3:**
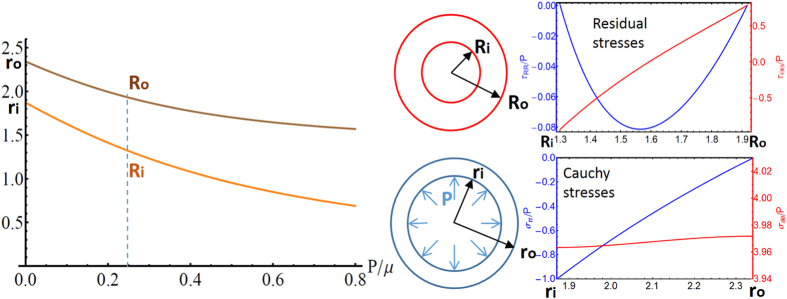
Functional adaptation in arteries: change of the unloaded geometry with the internal pressure *P* (left); resulting distribution of residual (right, top) and optimal Cauchy stresses (right, bottom). We set *r*_*o*_ = 2.34 mm, *r*_*i*_ = 1.87 mm, *P*/*μ* = 2.38 from the experimental data in^38^, giving *R*_*o*_ = 1.92 mm and *R*_*i*_ = 1.3 mm.

Let us consider an artery having internal and external spatial radii, *r*_*i*_ and *r*_*o*_ respectively, both much smaller than the vessel length, so that plane strain assumptions apply. Using a cylindrical coordinate system (*r*, *θ*), the equilibrium equation is (*σ*_*rr*_ *r*)_,*r*_ = *σ*_*θθ*_, with boundary conditions *σ*_*rr*_(*r*_*o*_) = 0 and *σ*_*rr*_(*r*_*i*_) = −*P*, with *P* being the blood pressure. Using a conjecture originally related only to the hoop stress component^24^,^25^, we assume that homeostasis for arteries corresponds to an optimal physical state characterized by the minimum stress gradient within the tissue. From a biological viewpoint, this hypothesis resides in the ability of the vascular smooth muscle cells to actively contract in response to the local stress level^13^. Accordingly, such a target state can be found by transforming the boundary elastic problem into a variational problem by minimizing the following functional:


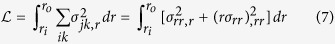


with 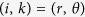, under the constraint 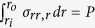 given by the boundary conditions. The solution for the homeostatic stress is given by solving the Euler-Lagrange equations arising from 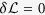, as detailed in the Supplementary Material and depicted in Fig. 3. We highlight that the resulting Cauchy stress has an optimal transmural gradient which is lower than the one obtained using an opened ring as a virtual unstressed state. We now assume that this target homeostatic state can be reached by the artery thanks to a functional adaptation process, i.e. undergoing a microstructural remodelling of the residually stressed configuration. In particular, let *R*_*o*_ and *R*_*i*_ be the outer and inner radii of such an optimal reference configuration; the global incompressibility condition imposes:


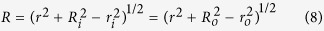


where *R* = *R*(*r*) is the material radial coordinate, such that the deformation gradient reads 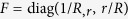. Taking into account the initial stress symmetry in the proposed constitutive symmetry, the residual stresses can be expressed as:


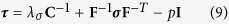


where 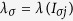, *I*_*σj*_ being the principal invariants of the optimal ***σ***. Considering that the equilibrium of the axisymmetric residually stressed configuration imposes 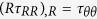, by virtue of the zero boundary conditions we obtain:


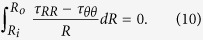


Substituting Eqs. (8, 9) in Eq. (10), we can determine the residually stressed configuration, i.e the value of *R*_*i*_, *R*_*o*_ and the corresponding ***τ***, that optimizes the stress gradient distribution in response to an internal blood pressure *P*. Such results are depicted in Fig. 3, describing how the artery mechanically adapts to the internal pressure by remodelling its residually stressed configuration.

**Figure 4:**
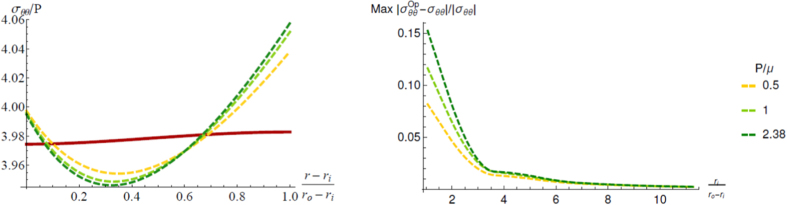
Comparison of the results of the proposed model (solid lines) and the opening angle method (dashed lines): distribution of the Cauchy hoop stress (left); and maximum stress difference as a function of the aspect ratio *r*_*i*_(*r*_0_ − *r*_*i*_) (right). As in Fig. 3, we set *r*_*o*_ = 2.34 mm, *r*_*i*_ = 1.87 mm, whilst we show the corresponding curves for *P*/*μ* = 0.5, 1, and 2.38.

Thus, the proposed model provides a more accurate description of the resulting Cauchy stresses compared to the approach based on the opening angle. In Fig. 4 (left), the optimal hoop stress distribution of our model (solid line) is depicted against the corresponding values obtained using the opening angle method for different values of *P*/*μ* (dashed lines). Using the same model parameters as in Fig. 4, it is shown that the opening angle method increasingly overestimates the maximum stress inside the material as *P*/*μ* increases. The two methods clearly coincide for *P* = 0, corresponding to an unstressed residual configuration. The maximum stress difference between the opening angle method and our model also increases when decreasing the aspect ratio 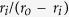 of the tube, as shown in Fig. 4 (right). Thus, we prove that the results of the proposed method are significantly more accurate than the ones of the standard method, especially when considering thick tubes subjected to a high magnitude of residual stresses.

## Experimental Determination of Residual Stresses by Wave Propagation

The proposed constitutive model for pre-stressed materials opens the path to new approaches for the non-destructive determination of the residual tensions. In particular, here we investigate if the residual stress components can be inferred by inverse-analysis from experimental measurements on waves on the undeformed, residually stressed body.

In the following, we provide an illustrative example dealing with infinitesimal wave propagation in an undeformed tubular tissue with residual stresses. Let us consider an inhomogeneous infinitesimal displacement **u** superposed on the undeformed, pre-stressed configuration, reading:


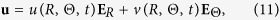


where **E**_*R*_ and **E**_Θ_ are the radial and tangential unit vectors, so that *u*, *v* represent the incremental radial and hoop motion fields, respectively. Indicating with **Γ** = Grad **u** the spatial displacement gradient associated with the incremental deformation, the incremental incompressibility condition and the incremental equations of motion read^26^:


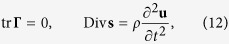


with the stress-free incremental boundary conditions **E**_*R*_**s** = **0** at the inner and outer radii, *R*_*i*_ and *R*_*o*_, respectively. The components of the incremental nominal stress **s** for the constitutive theory in Eq. (2) simplify as:


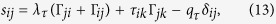


where *q*_*τ*_ is the incremental Lagrange multiplier p, and *λ*_*τ*_ is the real root of 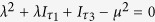, which is the equivalent of Eq. (6) in plane strain conditions.

**Figure 5:**
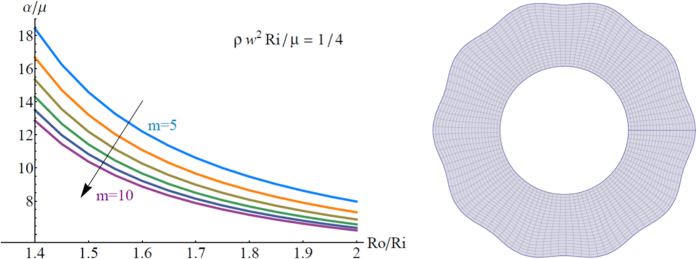
Left: Dispersion curves showing the pre-stress parameter *α*/*μ* for which a cylindrical wave with a given frequency *ω* can propagate as a function of the aspect ratio *R*_0_/*R*_*i*_ of the tube. We depict curves for different values of the angular wavenumber *m*, setting 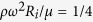. Right: Resulting wave shape for *R*_*o*_/*R*_*i*_ = 2 and *m* = 10. The amplitude is arbitrarily set as 0.15 *R*_*o*_ for the sake of graphical clarity.

Let us now make an educated guess of the solution and look for a time-harmonic cylindrical wave, whose displacement and stress components read:


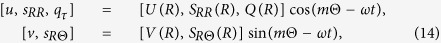


where *m* is the integer angular wavenumber, *ω* is the angular frequency, and the amplitudes 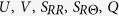 are scalar functions of *R* only. Following^27^, we introduce a functional relation between the incremental traction 
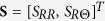
 and the displacement vector **U** = [*U*, *V*]^*T*^ as *R***S**(*R*) = **Z**(*R*)**U**(*R*), where **Z** is a *surface impedance matrix*. Using a standard method in elastodynamics^28^, the incompressibility condition and the equation of motion in Eq. (12) can be recast into a differential Riccati equation for **Z**:


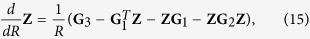


where **G**_1_, **G**_2_, **G**_3_ are the sub-blocks of the Stroh matrix, whose components can be found in the Supplementary Material. Eq. (15) can be solved numerically after assuming a given functional dependence for the residual stresses. Thus it is possible in principle to establish an inverse analysis for determining the residual stress distribution within a pre-stressed tube using wave propagation experiments. An illustrative example is sketched in the following. From Eq. (1), a simple expression of residual stress distribution is given by:


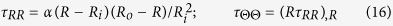


so that *α*/*μ* is a dimensionless parameter that defines the intensity of the pre-stresses. Using the stress-free boundary conditions and the functional form in Eq. (16) it is possible to numerically integrate Eq. (15) from the initial condition **Z** = **Z**(*R*_*o*_) = **0**, proving the existence of a time-harmonic cylindrical wave when the target condition det **Z**(*R*_*i*_) = **0** is met. In Fig. 5 (left), we depict the pre-stress parameter *α/μ* for which a cylindrical wave with a given frequency *ω* can propagate for different values of the angular wavenumber *m*, as a function of the aspect ratio of the tube. The resulting wave shape for *R*_*o*_ = 2*R*_*i*_ and *m* = 10 is finally shown in Fig. 5 (right). Accordingly, performing experiments using the propagation of time-harmonic cylindrical waves in pre-stressed tubes allows to unravel the underlying distribution of residual stresses. In practice, we can emit a wave at a given frequency *ω*, and then measure its speed *c* on the outer face at radius *R*_*o*_. The experimental measure of *c* allows to calculate the angular wavenumber *m*, since they are linked by the relation *m* = *ωR*_*o*_/*c*. Then by varying the frequency and measuring the speed, we can derive an experimental *ω*-*m* curve, which can be compared to the theoretical one, obtained for different initial distributions of the pre-stresses. This simple procedure finally could be the basis for establishing a nonlinear inverse-analysis that determines the residual stress distribution in a non-destructive way.

## Concluding Remarks

In summary, we have proposed a novel constitutive theory to describe the behaviour of residually stressed elastic materials. In contrast to previous models, which considered a virtual stress-free state without any physical counterpart, our approach defines a free energy explicitly depending on the quasi-static distribution of the residual stresses within the reference configuration. Imposing physical compatibility and symmetry arguments, we derived a novel class of constitutive behaviour, proving a generalization for a pre-stressed neo-Hookean material. This novel theory was later applied to the study of the functional adaptation of the artery to the internal blood pressure. In particular, our results are consistent with the ones obtained using the opening angle method, yet our optimal solution is energetically more favourable, therefore physically more relevant. Our proposed theory applies to those cases in which a steady distribution of residual stresses pre-exists within the biological material, so that an elastic free energy can be assumed. Nonetheless, it does not account for the microstructural rearrangement processes, such as cell duplication and/or migration, which provoke either the formation of residual stresses or the fluid -like stress relaxation phenomena^29^, which may be relevant up to the timescale of days^30^. Thus, future refinements will focus on introducing methods of individual cell-based models in order to capture the microscopic processes at the grain cell scale^31^. In another example, we have shown how the proposed model opens new ways for the non-destructive determination of the residual tension within living matter using wave propagation. Developing these non-destructive methods could improve our understanding of the physical mechanisms that regulate biological growth^19^.

Possible extensions of this preliminary exploration are multiple. Hence, the strain energy (5) can be made to depend on more invariants, including for instance a dependance on 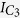 to reflect compressibility, see^32^ for an example. Intrinsic anisotropy can also be accounted for in the strain energy in order to capture the stiffening effect of the oriented bundles of type I collagen often encountered in soft tissues, see for example^33^. Similarly, the physical set-up can include non-homogeneity, to reflect the layered structure of most soft tissues. Finally, for the motions of real biological tissues, our hyperelastic model can be used to yield the elastic foundation of a constitutive relation including visco-elastic terms, see e.g.^34^.

## Additional Information

**How to cite this article**: Ciarletta, P. *et al*. On residual stresses and homeostasis: an elastic theory of functional adaptation in living matter. *Sci. Rep*. **6**, 24390; doi: 10.1038/srep24390 (2016).

## Supplementary Material

Supplementary Information

## Figures and Tables

**Figure 1:**
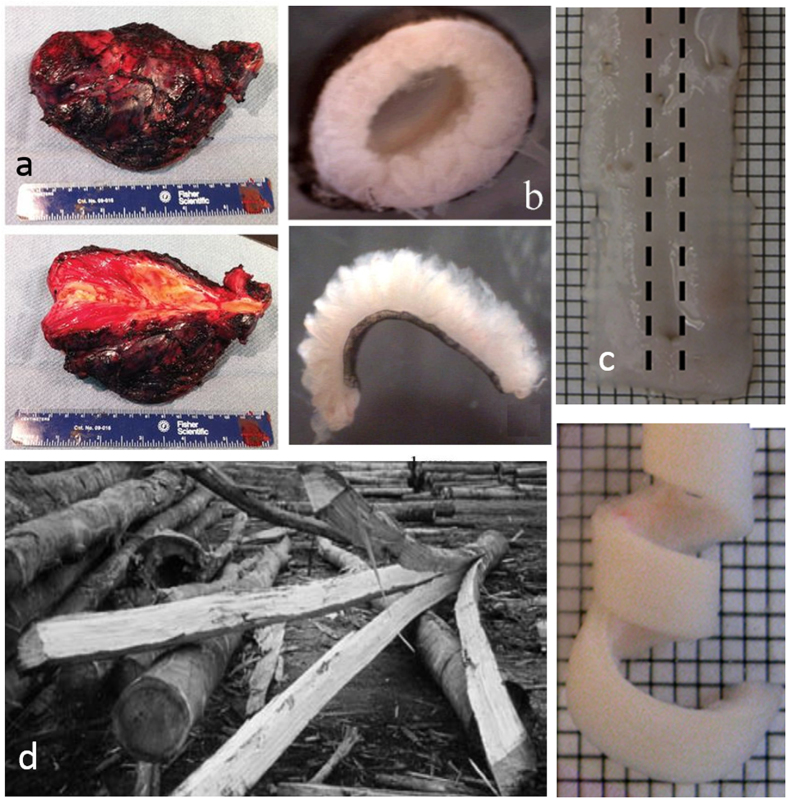
Release of residual stresses after cutting experiments in different living materials: solid tumour ((**a**) adapted from^14^, freely available online through the PNAS open access option); small intestine ((**b**) adapted from^35^, distributed under the terms of the Creative Commons Attribution License (http://creativecommons.org/licenses/by/2.0)); artery ((**c**) adapted from^36^, this image is not covered by the CC by license); *Eucalyptus* log ((**d**) adapted from^37^). All images are reprinted with permission of the copyright holders.
