# Supplementary material for: On residual stresses and homeostasis: an elastic theory of functional adaptation in living matter
Source: Sci Rep. 2016 Apr 26;6:24390. doi: 10.1038/srep24390 (PMC4845028; doi:10.1038/srep24390)
Supplement: Supplementary Information [file srep24390-s1.pdf]

On residual stresses and homeostasis: an elastic theory of functional adaptation in living matter

P. Ciarletta[1,2,*], M. Destrade[3] and A.L. Gower[3,4]

[1] Sorbonne Universités, UPMC Univ Paris 06,
CNRS, UMR 7190, Institut Jean Le Rond d'Alembert,
F-75005 Paris, France

[2] MOX and Fondazione CEN, Politecnico di Milano,
P.za Leonardo da Vinci 32, 20133 Milano, Italy

[3] School of Mathematics, Statistics and Applied Mathematics,
National University of Ireland Galway,
University Road, Galway, Ireland

[4] School of Mathematics, University of Manchester,
Oxford road, Manchester, M13 9PL, UK

[*] Corresponding author: pasquale.ciarletta@polimi.it

Supplemental Material

1 Derivation of a constitutive equation for elastic bodies with residual stresses

Under the assumptions of a macroscopic length-scale at the tissue scale and a characteristic time-scale short enough to neglect dissipative effects on the residual stresses (e.g. ageing phenomena), it is possible to define an elastic free energy $\Psi = \Psi(\mathbf{F}, \boldsymbol{\tau})$. Since we neglect any material anisotropy within the material (e.g. fiber reinforcement), we postulate that Ψ is a scalar-valued function depending on ten invariants: the six principal invariants of $\mathbf{C}$ and $\boldsymbol{\tau}$, and the four combined invariants of $\mathbf{C}$ and $\boldsymbol{\tau}$. From standard thermo-mechanical arguments, the constitutive equation of the Cauchy stress can be expressed as:

$$\boldsymbol{\sigma} = \mathbf{F}\frac{\partial \Psi}{\partial \mathbf{F}}(\mathbf{F}, \boldsymbol{\tau}) = \mathbf{F}\left(\sum_{k=1}^{3} \frac{\partial \Psi}{\partial I_{Ck}}\frac{\partial I_{Ck}}{\partial \mathbf{F}} + \sum_{m=1}^{4} \frac{\partial \Psi}{\partial J_m}\frac{\partial J_m}{\partial \mathbf{F}}\right) \tag{S1}$$

where the chain differentiation rule has been used. Recalling the following tensor rules:

$$\frac{\partial I_{C1}}{\partial \mathbf{F}} = 2\mathbf{F}^T; \qquad \frac{\partial I_{C2}}{\partial \mathbf{F}} = 2I_{C1}\mathbf{F}^T + \mathbf{C}\mathbf{F}^T; \qquad \frac{\partial I_{C3}}{\partial \mathbf{F}} = 2I_{C3}\mathbf{F}^{-1} \tag{S2}$$

$$\frac{\partial J_1}{\partial \mathbf{F}} = 2\boldsymbol{\tau}\mathbf{F}^T; \quad \frac{\partial J_2}{\partial \mathbf{F}} = 2\boldsymbol{\tau}\mathbf{C}\mathbf{F}^T + 2\mathbf{C}\boldsymbol{\tau}\mathbf{F}^T; \quad \frac{\partial J_3}{\partial \mathbf{F}} = 2\boldsymbol{\tau}^2\mathbf{F}^T; \quad \frac{\partial J_4}{\partial \mathbf{F}} = 2\boldsymbol{\tau}^2\mathbf{C}\mathbf{F}^T + 2\mathbf{C}\boldsymbol{\tau}^2\mathbf{F}^T \tag{S3}$$

it is possible to derive the Equation (3) of the Letter. It is useful to highlight that the constitutive equation for a compressible material can easily be obtained by removing the Lagrange multiplier p and postulating a functional dependence of the free energy on I_{C3}.

2 Derivation of the initial stress symmetry conditions

The *initial stress symmetry* (ISS) enforces the necessity to recover the residually stressed configuration from the loaded state by reversing the deformation mapping:

$$\boldsymbol{\tau} = -p_\tau \boldsymbol{I} + \mathbf{F}^{-1}\frac{\partial \Psi}{\partial \boldsymbol{F}}(\mathbf{F}^{-1}, \boldsymbol{\sigma}) \tag{S4}$$

where p_τ is the Lagrange multiplier due to the incompressibility constraint. Hereafter we show that the ISS conditions in Eq. (S4) can be expressed as a set of scalar equations, relating the free energy density $\Psi(\boldsymbol{F}, \boldsymbol{\tau})$ and the invariants of $\boldsymbol{\tau}$ and $\mathbf{C}$. Let us first use Eq. (S4) to write the residual stress as

$$\begin{aligned}\boldsymbol{\tau} = -p_\tau \boldsymbol{I} + 2\Psi^{\sigma}_{,I_1}\boldsymbol{C}^{-1} + 2\Psi^{\sigma}_{,I_2}(I_2\boldsymbol{C}^{-1} - \boldsymbol{C}^{-2}) + 2\Psi^{\sigma}_{,J_1}\boldsymbol{F}^{-1}\boldsymbol{\sigma}\boldsymbol{F}^{-T} + 2\Psi^{\sigma}_{,J_3}\boldsymbol{F}^{-1}\boldsymbol{\sigma}^2\boldsymbol{F}^{-T} \\ + 2\Psi^{\sigma}_{,J_2}\boldsymbol{F}^{-1}(\boldsymbol{\sigma}\boldsymbol{B}^{-1} + \boldsymbol{B}^{-1}\boldsymbol{\sigma})\boldsymbol{F}^{-T} + 2\Psi^{\sigma}_{,J_4}\boldsymbol{F}^{-1}(\boldsymbol{\sigma}^2\boldsymbol{B}^{-1} + \boldsymbol{B}^{-1}\boldsymbol{\sigma}^2)\boldsymbol{F}^{-T}\end{aligned} \tag{S5}$$

where $\Psi^{\sigma}_{I_k} := \Psi_{,I_k}(\boldsymbol{F}^{-1}, \boldsymbol{\sigma})$, $\Psi^{\sigma}_{,J_m} := \Psi_{,J_m}(\boldsymbol{F}^{-1}, \boldsymbol{\sigma})$, $I_1 = \text{tr}(\boldsymbol{C})$, $I_2 = \text{tr}(\boldsymbol{C}^{-1})$ using the Cayley-Hamilton theorem with $\det \boldsymbol{C} = 1$, and comma denotes partial derivative with respect to the following term. The above must hold for every $\boldsymbol{\tau}$ and $\boldsymbol{C}$. Thus, we can substitute the constitutive equation for the Cauchy stress into Eq. (S5), obtaining :

$$\alpha_{ijkmn}\boldsymbol{C}^i\boldsymbol{\tau}^j\boldsymbol{C}^k\boldsymbol{\tau}^m\boldsymbol{C}^n = \mathbf{0}, \tag{S6}$$

where we assume summation for i, k, n over $\{-2, -1, 0, 1, 2\}$ and j, m over $\{0, 1, 2\}$. Dealing with symmetric tensors, we have the symmetries $\alpha_{ijkmn} = \alpha_{njkmi} = \alpha_{nmkji}$, where the values of α_{ijkmn} are not reported here for the sake of brevity.

Finally, it is possible to further simplify Eq. (S6) to obtain nine scalar equations that hold for any choice of Ψ and of all the invariants. This can be done by varying $\boldsymbol{C}$ and $\boldsymbol{\tau}$ while

keeping the invariants fixed. Since Eq.(S6) must hold for any $\boldsymbol{C}$ and $\boldsymbol{\tau}$, we obtain nine scalar equations which compactly rewrite

$$\boldsymbol{b} + \boldsymbol{P}^{\{1\}}\Psi^{\sigma}_{,J_1} + \boldsymbol{P}^{\{2\}}\Psi^{\sigma}_{,J_2} + \Psi_{,J_n}\left(\boldsymbol{P}^{\{3\}}_{\{n\}}\Psi^{\sigma}_{,J_3} + \boldsymbol{P}^{\{4\}}_{\{n\}}\Psi^{\sigma}_{,J_4}\right) = 0, \tag{S7}$$

$$\Psi_{J_n}\left(\boldsymbol{Q}^{\{1\}}_{\{n\}}\Psi^{\sigma}_{,J_1} + \boldsymbol{Q}^{\{2\}}_{\{n\}}\Psi^{\sigma}_{,J_2} + \boldsymbol{Q}^{\{3\}}_{\{n\}}\Psi^{\sigma}_{,J_3} + \boldsymbol{Q}^{\{4\}}_{\{n\}}\Psi^{\sigma}_{,J_4}\right) = 0, \tag{S8}$$

where n sums over $\{0,1,2,3,4\}$, $\Psi_{J_0} := 1$, and:

$$\boldsymbol{b} = \begin{bmatrix} 2\Psi^{\sigma}_{,I_2} \\ -2\Psi^{\sigma}_{,I_1} \\ p_{\mathcal{T}} \\ 0 \\ 0 \\ 1 \end{bmatrix}, \quad \boldsymbol{P}^{\{1\}} = 4\begin{bmatrix} \Psi_{I_2} \\ p/2 \\ -\Psi_{,I_1} \\ -2\Psi_{,J_2} \\ 2I_2\Psi_{,J_2} \\ -\Psi_{,J_1} - 2I_1\Psi_{,J_2} \end{bmatrix}, \quad \boldsymbol{P}^{\{2\}} = 4\begin{bmatrix} p \\ I_2 p - 2\Psi_{,I_1} - 2I_1\Psi_{,I_2} \\ 2\Psi_{,I_2} \\ 0 \\ -2\Psi_{,J_1} \\ -4\Psi_{,J_2} \end{bmatrix}, \tag{S9}$$

$$\boldsymbol{Q}^{\{1\}}_{\{1\}} = \boldsymbol{Q}^{\{1\}}_{\{2\}} = \boldsymbol{Q}^{\{2\}}_{\{1\}} = \boldsymbol{Q}^{\{2\}}_{\{2\}} = \boldsymbol{0}. \tag{S10}$$

The other matrices in Eq.(S7) have cumbersome expressions and will be not reported here for the sake of brevity. In summary, Eqs (S7) and (S8) represent respectively 6 and 3 scalar equations, respectively, determining the ISS conditions, the unknowns being the scalar functions Ψ, p and $p_{\mathcal{T}}$.

For the sake of simplicity, we now look for simplified expression neglecting the functional dependence on J_3 and J_4, which represent higher order terms in the mixed functional dependence. In this case, the fourth scalar equation of Eq. (S7) rewrites:

$$-8\Psi_{,J_2}\Psi^{\boldsymbol{\sigma}}_{,J_1} = 0 \tag{S11}$$

which is only possible, for every $\mathbf{F}$ and $\boldsymbol{\tau}$, if and only if Ψ does not depend on J_2 or on J_1. In particular, if Ψ does not depend also on J_2, then Eq. (S7) reduces to:

$$4\Psi_{,J_1}\Psi^{\boldsymbol{\sigma}}_{,J_1} = 1, \quad \frac{\Psi^{\boldsymbol{\sigma}}_{,I_2}}{\sqrt{\Psi^{\boldsymbol{\sigma}}_{,J_1}}} + \frac{\Psi_{,I_2}}{\sqrt{\Psi_{,J_1}}} = 0, \quad p\Psi^{\boldsymbol{\sigma}}_{,J_1} = \Psi^{\boldsymbol{\sigma}}_{,I_1}, \quad p_{\boldsymbol{\tau}}\Psi_{,J_1} = \Psi_{,I_1}, \tag{S12}$$

where we have used Eq. $(\mathrm{S12})_1$ to derive the other three equations. Let us now impose the physical compatibility of the strain energy, by letting $\boldsymbol{F} = \boldsymbol{I}$, $\boldsymbol{\sigma} = \boldsymbol{\tau}$ and $p_\tau = p$. We obtain that $\Psi_{,J_1} = 1/2$, $2\Psi_{,I_1} = p$ and $\Psi_{,I_2} = 0$ for $\boldsymbol{F} = \boldsymbol{I}$. Accordingly, we derive the class of free energies for residually stressed material expressed by Eq.(5) in our Letter. The generalization to incompressible neo-Hookean materials in Eq.(6) in our Letter is straightforward, thanks to the possibility to invert explicitly its constitutive relation in order to write the strain as a function of the Cauchy stress [1]. Finally, the proposed approach and the initial stress symmetry condition are also valid for compressible materials, with minor alterations introduced by the chosen functional dependence on $I_{C3} = det\mathbf{C}$.

3 Optimal solution for the Cauchy stress within an artery

The variational problem determining the optimal distribution of the Cauchy stress in an artery under internal pressure rewrites:

$$\delta\mathcal{L} = \delta\int_{r_i}^{r_o}[\sigma_{rr,r}^2 + (r\sigma_{rr})_{,rr}^2]dr = 0 \tag{S13}$$

under the constraint $\int_{r_i}^{r_o}\sigma_{rr,r}dr = P$ given by the boundary conditions. Before solving the corresponding Euler-Lagrange equations, let us introduce the following dimensionless

variables:

$$\varrho = \frac{r - r_i}{r_o - r_i} \quad \text{and} \quad \varsigma(\rho) = \frac{\sigma_{rr}(r)}{P}, \tag{S14}$$

so that

$$\sigma_{rr,r} = \varsigma' \frac{P}{r_o - r_i}, \quad \sigma_{rr,rr} = \varsigma'' \frac{P}{r_o - r_i{}^2} \tag{S15}$$

and

$$\sigma_{\theta\theta,r} = \frac{P}{r_o - r_i} \left(2\varsigma' + (\rho + \alpha)\varsigma''\right) \tag{S16}$$

where we have introduced $\alpha = r_i/(r_o - r_i)$. The functional $\mathcal{L}$ in Eq.(S13) therefore rewrites :

$$\mathcal{L} = \frac{P^2}{r_i^2}\alpha^2 \int_0^1 [(\varsigma')^2 + (2\varsigma' + (\varrho + \alpha)\,\varsigma'')^2] d\varrho \tag{S17}$$

with the constraint

$$\varsigma(1) = 0 \ \text{ and } \ \varsigma(0) = -1 \implies \int_0^1 \varsigma' d\varrho = 1. \tag{S18}$$

Using the calculus of variations [2], we find the following Euler-Lagrange equation

$$\frac{\partial f}{\partial \varsigma'} - \frac{d}{d\varrho}\frac{\partial f}{\partial \varsigma''} = \Lambda \ \text{ for } \varrho \in (0,1) \quad \text{and} \quad \frac{\partial f}{\partial \varsigma''} = 0 \ \text{ for } \varrho = 0, 1 \tag{S19}$$

where

$$f = (\varsigma')^2 + (2\varsigma' + (\varrho + \alpha)\,\varsigma'')^2 \tag{S20}$$

and Λ is a Lagrange multiplier that appears due to the constraint (S18). The solution of (S19) is given by

$$\varsigma' = \frac{\Lambda}{6} - \frac{\Lambda}{6}(\sqrt{13} - 3)\frac{\alpha^{\frac{\sqrt{13}}{2}+\frac{1}{2}} - (1+\alpha)^{\frac{\sqrt{13}}{2}+\frac{1}{2}}}{\alpha^{\sqrt{13}} - (1+\alpha)^{\sqrt{13}}}(\alpha + \varrho)^{\frac{\sqrt{13}}{2}+\frac{1}{2}} + \frac{2\Lambda}{3(\sqrt{13}-3)} \frac{\alpha^{\frac{\sqrt{13}}{2}-\frac{1}{2}} - (1+\alpha)^{\frac{\sqrt{13}}{2}-1\frac{1}{2}}}{\alpha^{\sqrt{13}} - (1+\alpha)^{\sqrt{13}}} \left(\frac{\alpha(1+\alpha)}{\alpha + \varrho}\right)^{\frac{\sqrt{13}}{2}+\frac{1}{2}} \tag{S21}$$

Finally, we determine Λ from the constraint (S18) to be

$$\Lambda = 6\Big(\int_0^1 1 - (\sqrt{13}-3)\frac{\alpha^{\frac{\sqrt{13}}{2}+\frac{1}{2}} - (1+\alpha)^{\frac{\sqrt{13}}{2}+\frac{1}{2}}}{\alpha^{\sqrt{13}} - (1+\alpha)^{\sqrt{13}}}(\alpha+\varrho)^{\frac{\sqrt{13}}{2}+\frac{1}{2}} + \frac{4}{\sqrt{13}-3}\frac{\alpha^{\frac{\sqrt{13}}{2}-\frac{1}{2}} - (1+\alpha)^{\frac{\sqrt{13}}{2}-1\frac{1}{2}}}{\alpha^{\sqrt{13}} - (1+\alpha)^{\sqrt{13}}}\left(\frac{\alpha(1+\alpha)}{\alpha+\varrho}\right)^{\frac{\sqrt{13}}{2}+\frac{1}{2}} d\varrho\Big)^{-1}. \quad \text{(S22)}$$

Finally, we compare our optimal solution for the Cauchy stress against the one obtained using a opened ring as virtual state, also known as the *opening angle method* [3], as depicted in Figure 1.

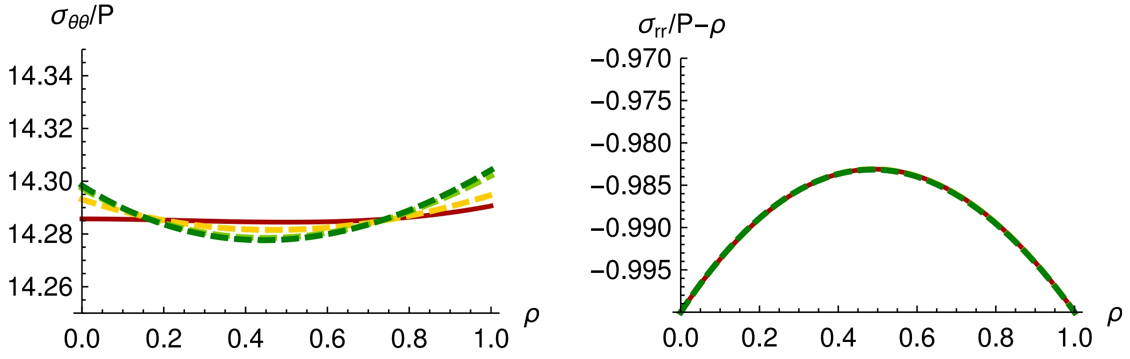

Figure 1: The curves of the dimensionless Cauchy stress against the dimensionless radius ρ for the opening angle method (dashed lines) and our optimal stress (red solid lines). We set physically relevant parameters for the descending thoracic aorta: $r_i = 20\,\text{mm}$, $r_o = 21.4\,\text{mm}$, so that $\alpha^{-1} = 0.07$. As the dashed curves shade from yellow to green P/μ goes through the values 0.05, 0.2 and 0.35 with optimal opening angle $\phi = 65.9°$, 204.2° and 261.723°; stress free reference inner radius 20.3 mm, 25.2 mm and 31.1 mm; and stress free reference outer radius 22 mm, 27.8 mm and 34.4 mm respectively.

We highlight that $\sigma_{\theta\theta}$ for the opening angle method tends to the optimal stress $\sigma_{\theta\theta}$ only if P/μ tends to zero, while the plots for σ_{rr} do not show significant differences between the two methods.

Wave propagation in a residually stressed tube

Let us deal with the infinitesimal wave propagation of an undeformed tubular tissue with residual stresses. We consider an inhomogeneous infinitesimal wave $\mathbf{u}$ of the form:

$$\mathbf{u} = u(R, \Theta, t)\mathbf{e}_R + v(R, \Theta, t)\mathbf{e}_\Theta, \tag{S23}$$

where $\mathbf{E}_R$ and $\mathbf{E}_\Theta$ are the radial and tangential unit vectors, so that u, v represent the incremental radial and hoop displacement fields, respectively. Indicating with $\mathbf{\Gamma} = \mathrm{Grad}\,\mathbf{u}$ the spatial displacement gradient associated with the incremental deformation, the incremental incompressibility condition reads:

$$\mathrm{tr}\,\Gamma = 0 \tag{S24}$$

Following the incremental elastic theory [4], the incremental equations of motion read:

$$\mathrm{Div}\,\mathbf{s} = \rho \frac{\partial^2 \mathbf{u}}{\partial t^2} \tag{S25}$$

with boundary conditions $\mathbf{e}_R \mathbf{s} = 0$ at the inner and outer radii, R_i and R_o, respectively. The components of the incremental nominal stress $\mathbf{s}$ for the constitutive theory in Eq.(5) of the article read:

$$s_{ij} = \lambda_\tau(\Gamma_{ji} + \Gamma_{ij}) + \tau_{ik}\Gamma_{jk} - q_\tau \delta_{ij}, \tag{S26}$$

where q_τ is the incremental pressure, and λ_τ is the real root of $\lambda^2 + \lambda I_{\tau_1} + I_{\tau_3} - \mu^2 = 0$, which is the equivalent of Eq.(6) of the article in plane strain conditions.

Let as now make an educated guess of the solution in the form of a time-harmonic cylin-

drical wave, having displacement and stress components defined as:

$$
\begin{aligned}
[u, s_{RR}, q_\tau] &= [U(R), S_{RR}(R), Q(R)] \cos(m\Theta - \omega t), \\
[v, s_{R\Theta}] &= [V(R), S_{R\Theta}(R)] \sin(m\Theta - \omega t),
\end{aligned} \tag{S27}
$$

where m is the integer angular wavenumber, ω is the angular frequency, and the amplitudes $U, V, S_{RR}, S_{R\Theta}, Q$ are scalar functions of R only. Following [5], the incompressibiity condition Eq.(S24) and the equation of motion Eq.(S25) can be recast in a system of four ordinary differential equation of the first order:

$$
\frac{d}{dR}\begin{bmatrix} \mathbf{U} \\ R\mathbf{S} \end{bmatrix} = \frac{1}{R}\begin{bmatrix} \mathbf{G}_1 & \mathbf{G}_2 \\ \mathbf{G}_3 & -\mathbf{G}_1^T \end{bmatrix}\begin{bmatrix} \mathbf{U} \\ R\mathbf{S} \end{bmatrix} \quad \text{with} \quad \begin{cases} \mathbf{U} = [U(R), V(R)]^T, \\ \mathbf{S} = [S_{RR}(R), S_{R\Theta}(R)]^T, \end{cases} \tag{S28}
$$

also known as the Stroh formulation of the incremental problem. The sub-blocks of the Stroh matrix in Eq.(S28) have the following components

$$
\mathbf{G}_1 = \begin{bmatrix} -1 & -m \\ \frac{mR\lambda_\tau}{f+R\lambda_\tau} & \frac{\lambda_\tau}{\tau_{RR}+\lambda_\tau} \end{bmatrix}, \qquad \mathbf{G}_2 = \begin{bmatrix} 0 & 0 \\ 0 & \frac{1}{\tau_{RR}+\lambda_\tau} \end{bmatrix},
$$

$$
\mathbf{G}_3 = \begin{bmatrix} 4\lambda_\tau - \rho R^2\omega^2 + (1+m^2)\tau_{\Theta\Theta} + \frac{\tau_{RR}[\tau_{RR}+(1+m^2)\lambda_\tau]}{\tau_{RR}+\lambda_\tau} & m\left[4\lambda_\tau + 2\tau_{\Theta\Theta} + \frac{\tau_{RR}(\tau_{RR}+2\lambda_\tau)}{\tau_{RR}+\lambda_\tau}\right] \\ m\left[4\lambda_\tau + 2\tau_{\Theta\Theta} + \frac{\tau_{RR}(\tau_{RR}+2\lambda_\tau)}{(\tau_{RR}+\lambda_\tau)}\right] & 4m^2\lambda_\tau + (1+m^2)I_{\tau 1} - \frac{\tau_{RR}^2}{(\tau_{RR}+\lambda_\tau)} - \rho R^2\omega^2 \end{bmatrix}, \tag{S29}
$$

Let us now introduce a functional relation between the incremental traction and the displacements vectors as $R\,\mathbf{S}(R) = \mathbf{Z}(R)\,\mathbf{U}(R)$, where $\mathbf{Z}$ is a *surface impedance matrix* [6]. Substituting the previous expression into Eq.(S28), we derive the following differential Riccati equation for $\mathbf{Z}$,

$$
\frac{d}{dR}\mathbf{Z} = \frac{1}{R}\left(\mathbf{G}_3 - \mathbf{G}_1^T\mathbf{Z} - \mathbf{Z}\mathbf{G}_1 - \mathbf{Z}\mathbf{G}_2\mathbf{Z}\right), \tag{S30}
$$

Let us now clarify how Eq.(S30) can be used to establish a non-destructive method for measuring the residual stress distribution within a pre-stressed tube. An illustrative example is sketched in the following. Imposing the equilibrium equations in the undeformed configuration, a simple expression of residual stress distribution is given by:

$$\tau_{RR} = \alpha(R - R_i)(R_o - R)/R_i^2; \qquad \tau_{\Theta\Theta} = (R\tau_{RR})_{,R} \tag{S31}$$

Using the stress-free boundary conditions and the functional form in Eq.(S31) it is possible to numerically integrate Eq. (S30) from the initial condition $\mathbf{Z} = \mathbf{Z}(R_i) = \mathbf{0}$ (resp. $\mathbf{Z} = \mathbf{Z}(R_o) = \mathbf{0}$) , proving the existence of a time-harmonic cylindrical wave when the target condition $\det \mathbf{Z}(R_o) = \mathbf{0}$ (resp.$\det \mathbf{Z}(R_i) = \mathbf{0}$) is met.

The Hamiltonian structure and algebraic properties of the Stroh matrix yield a robust numerical procedure to determine when cylindrical waves appear on either of the faces of the residually stressed tube. In particular, we found the unique, symmetric, semi-definite solution of the differential Riccati equation for $\mathbf{Z}$ in Eq.(S30) by numerical integration using the software *Mathematica* (Wolfram Inc., version 10.1, Champaign, IL) once τ_{RR}, ω, R_o and R_i are prescribed. Thus, for a given tube, we adjust the pre-stress parameter α/μ, proportional to the amplitude of the residual stress, until we meet the target condition for a given m. Once α/μ is determined, we integrate the first line of Eq.(S28), i.e.

$$\frac{d\mathbf{U}}{dR} = \frac{1}{R}\mathbf{G}_1\mathbf{U} + \frac{1}{R}\mathbf{G}_2\mathbf{Z}\mathbf{U}, \tag{S32}$$

simultaneously with Eq.(S30) to compute the incremental wave field throughout the thickness of the tube wall.

References

[1] Destrade, M, Murphy, JG and Saccomandi, G. 2012. *Simple shear is not so simple.* Int J Nonlinear Mech 47(2), 210-214

[2] Dacorogna, B. *Direct methods in the calculus of variations.* Springer Science & Business Media, 2007.

[3] Chuong, CJ and Fung, YC. 1986. *Residual Stress in Arteries.* In: Frontiers in Biomechanics,117-129.

[4] Ogden, R.W. 1997. *Nonlinear elastic deformations.* Dover Publications.

[5] Shuvalov,A.L. 2003. *A sextic formalism for threedimensional elastodynamics of cylindrically anisotropic radially inhomogeneous materials.* Proceedings of the Royal Society of London A: Mathematical, Physical and Engineering Sciences 459, 2035: 1611-1639, 2003.

[6] Biryukov,S.V. 1985. *Impedance method in the theory of elastic surface wave.* Sov. Phys. Acoust. 31: 350354.
